# Inactivation of *Bacillus cereus* Spores and Vegetative Cells in Inert Matrix and Rice Grains Using Low-Pressure Cold Plasma

**DOI:** 10.3390/foods13142223

**Published:** 2024-07-15

**Authors:** María Inés Valdez-Narváez, M. Teresa Fernández-Felipe, Antonio Martinez, Dolores Rodrigo

**Affiliations:** Instituto de Agroquímica y Tecnología de Alimentos (IATA-CSIC), 46980 Paterna, Valencia, Spain; mavalna31@iata.csic.es (M.I.V.-N.); mteresa.fer@iata.csic.es (M.T.F.-F.); amartinez@iata.csic.es (A.M.)

**Keywords:** low-pressure cold plasma, *B. cereus*, spore, rice, Weibull model

## Abstract

This study investigated the effects of low-pressure cold plasma on the inactivation of *Bacillus cereus* vegetative cells and spores in an inert matrix (borosilicate glass slide) and in rice grains, using oxygen as ionization gas. Greater reductions in *B. cereus* counts were observed in vegetative cells rather than spores. The experimental data obtained show that both the power of the plasma treatment and the matrix proved to be determining factors in the inactivation of both the spores and vegetative cells of *B. cereus*. To characterize the inactivation of *B. cereus*, experimental data were accurately fitted to the Weibull model. A significant decrease in parameter “a”, representing resistance to treatment, was confirmed with treatment intensification. Furthermore, significant differences in the “a” value were observed between spores in inert and food matrices, suggesting the additional protective role of the food matrix for *B. cereus* spores. These results demonstrate the importance of considering matrix effects in plasma treatment to ensure the effective inactivation of pathogenic microorganisms, particularly in foods with low water activity, such as rice. This approach contributes to mitigating the impact of foodborne illnesses caused by pathogenic microorganisms.

## 1. Introduction

The cultivation of rice (*Oryza sativa* L.) holds historical significance as one of the oldest practices, considering it as a staple food for humans and its global prominence as one of the most extensively cultivated crops. In recent decades, rice production has witnessed nearly a twofold increase, facing threats from various challenges, such as limited agricultural land, water scarcity, soil fertility concerns, climate change, insect infestations, and diseases [1,2]. *B. cereus* is one of the main contaminants of rice. Although rice is usually cooked at 100 °C before consumption, spores of this micro-organism can survive, posing a health risk to consumers [3].

*B. cereus* is a Gram-positive bacterium known for its ability to form spores, which allows it to survive in diverse environmental conditions. This bacterium is commonly found in soil and can contaminate various types of food, causing foodborne illnesses [4,5]. Diarrheal syndrome is an infection caused by the growth of the microorganism in the small intestine, resulting in diarrhea, while emetic syndrome is a syndrome that results in vomiting when food containing the previously formed cereulide toxin is ingested. *B. cereus* can be present in a wide range of foods, including rice, pasta, meat, vegetables, and dairy products. The spores of this bacterium are resistant to heat, allowing them to survive cooking processes [6]. Therefore, proper food handling, cooking, and storage are essential to prevent the spores’ germination, the growth of *B. cereus*, and the production of toxins [4,5]. In the European Union, the One Health 2022 zoonoses report [7] indicated that *B. cereus* toxins are ranked as the leading cause of reported Foodborne Outbreaks (FBOs) attributed to bacterial toxins. Specifically, there was a significant surge in the number of adverse events (AEs) caused by *B. cereus* toxins in 2022 compared to 2021 (with an increase of 219 AEs in 2022, representing a relative rise of 251.7%). *B. cereus* toxins were identified as the causative agent in five highly significant adverse event outcomes (AEOs), defined as having 100 or more reported cases. In this same report, the EFSA associated 8.33% of outbreaks with non-animal-origin processed foods, including rice.

In the quest to address foodborne illnesses, heat sterilization has been employed as the primary choice in the food industry. Nevertheless, traditional heat processes can result in a decline in the quality of food, particularly in cases involving heat-sensitive products. In recent times, non-thermal technologies have been implemented for the microbial management of food, focusing notably on spore inactivation [8], and this is where the plasma, as a non-thermal technology that has the ability to inactivate microbial spores [8], plays a crucial role in this study. Plasma is considered the fourth state of matter; it has unique properties that distinguish it from the following three basic states: solids, liquids, and gases. It is generated via atoms that have undergone ionization, meaning that they have lost or gained electrons, resulting in a mixture of free electrons and ions [9,10]. This characteristic makes plasma an excellent electrical conductor and gives it distinctive properties that make it valuable in various applications. The disinfection effect of plasma technology is due to the number of reactive species that it can generate, including mainly reactive oxygen (ROS), nitrogen (RNS) species, and UV radiation. Reactive oxygen and nitrogen substances include O_3_, H_2_O_2_, OH, O_2_^¯^, and NO_2_ specifically, which can cause significant oxidative damage to bacterial cells, resulting in oxidative stress and ultimately leading to microbial death [11]. Various types of plasma can be generated depending on temperature and pressure conditions [9]. This study focuses on low-temperature (<70 °C) and low-pressure plasma, which is characterized because it is easier to control the working conditions [12,13].

Research has been carried out to understand how different parameters, such as power, time, frequency, voltage, plasma composition, temperature, or plasma electrode configurations, affect plasma generation and, therefore, the efficiency of microbial inactivation [14,15,16]. However, in addition to these electrical parameters, food matrices have also been found to play a crucial role in the efficiency of microbial inactivation. For example, recent research has shown that food composition and moisture can significantly influence responses to plasma treatment [16,17]. Another relevant aspect is the variability in the microorganism’s sensitivity to plasma depending on the microbial species but also on the vegetative forms (vegetative cells or spores), underscoring the need to investigate and understand the specific interactions between the plasma and microorganisms of interest [17,18,19].

Another aspect to consider in cold plasma technology is the transition of this technology from the laboratory to the food industry since this also represents a challenge. Therefore, modeling the outcome of microbial inactivation becomes essential. Mathematical modeling allows plasma treatment processes to be optimized according to specific processing parameters, the food matrix, and target microorganisms. By understanding how these factors interact, more effective strategies can be developed to ensure food safety.

In this context, the aim of this study was to evaluate the impact of low-pressure cold plasma on *B. cereus* vegetative cells and spores in different matrices. Furthermore, data can be fitted to a mathematical model to improve understanding of the potential applications of cold plasma treatment, particularly in the realms of microbial control and food safety on an industrial scale.

## 2. Materials and Methods

### 2.1. Matrix

Two types of matrices were employed in this study. A borosilicate glass slide (24 × 50 mm), as an inert and non-porous surface, is a model for food contact surfaces that enables the observation of the direct impact of cold plasma on the microorganism without external influences, and round grain rice (*Oryza sativa* L.) (5 g) bought from a local supermarket, can be used as a model for low water activity food, allowing the evaluation of cold plasma in real food environments. Prior to the plasma treatment, the moisture content of the rice was determined, and it was dried at 80 °C for 24 h to a constant weight, reaching a moisture content of 9%.

### 2.2. Test Microorganism

The tests were conducted using a pure lyophilized culture of *B. cereus* obtained from the Spanish Type Culture Collection (CECT 148), (Valencia-Spain). 

The lyophilized culture was reconstituted with 0.2 mL of a sterile nutrient broth (NB) liquid medium (Scharlab Chemie S.A., Barcelona, Spain). After a 30 min incubation period at 30 °C, the entire suspension was inoculated into an Erlenmeyer flask containing 500 mL of NB medium. The flask was incubated at 30 °C in a thermostatic bath with continuous shaking for 14 h to achieve cells in a stationary growth phase. Subsequently, the cells underwent two centrifugation steps at 3752× *g* at 4 °C for 15 min using a Beckman centrifuge (JLA-16,250 rotor) (Brea, CA, USA). After decanting the supernatant, the cells were resuspended in 50 mL of NB medium. After the second centrifugation, the cells were again resuspended in NB and distributed into 2 mL cryovials with 1 mL per cryovial. To each cryovial, 1 mL of 20% glycerol in NB, serving as a cryoprotectant, was added. The 2 mL samples were promptly frozen and stored at −80 °C until needed. The concentration of *B. cereus* in the cryovials was determined by plate count, revealing a concentration of 10^8^ CFU/mL.

### 2.3. Sporulation Procedure

The *B. cereus* in cryovials from Section 2.2 was used for bacterial sporulation. The strain was reactivated in nutrient broth, undergoing shaking for 24 h at 32 °C. Subsequently, 20 Roux flasks (Fisher Scientific SL, Madrid, Spain) containing fortified nutrient agar (Scharlab, Barcelona, Spain) were prepared; each flask had a 0.5 mL inoculum of the *B. cereus* culture, and they were incubated at 30 °C.

Once the sporulation level reached approximately 90%, spores were collected using a modified Digralsky metal loop (Deltalab, Barcelona, Spain). This involved gently sweeping the agar surface and washing it with double-distilled water. The collected solution was centrifuged at 2500× *g* for 15 min at 5 °C, and the supernatant was removed. The spores were then re-suspended in 5 mL of double-distilled water and subjected to centrifugation under the same conditions. This process was repeated four times. Finally, the spores from the pellet were stored at 4 °C in distilled water.

### 2.4. Plasma Equipment

A low-pressure cold plasma system based on a Dielectric Barrier Discharge (DBD) design from Electronic Diener Plasma Surface Technology PCCE, model Pico-AR-200-PCCE7, was used. This system generates plasma through two circular plate electrodes and operates under low-pressure conditions (0.35 mbar), facilitated by a vacuum pump (Leybold Trivac D16T, Barcelona, Spain). This equipment operates at a frequency of 13.56 MHz and a power range of 0 to 300 W.

In this study, 100% pure oxygen gas (Alphagaz) was employed as the ionization gas, and the system operated at a pressure of 0.35 mbar. Power levels of 100, 200, and 300 W were applied, with treatment durations ranging from 5 to 60 min as a variable parameter in the process.

### 2.5. Plasma Treatment

Before each plasma treatment, different samples were prepared. For spores, borosilicate glass slides, previously disinfected and degreased, were inoculated with 100 µL of *B. cereus* spores at a concentration of 10^7^ CFU/mL. Similarly, 5 g of rice were weighed separately, introduced in a borosilicate crystallization capsule (diameter 6 cm), and inoculated with 100 µL of *B. cereus* spores at a concentration of 10^7^ CFU/g. In both cases, before plasma treatment, the samples were dried in a biosafety cabinet at room temperature for 20 h. For vegetative cells, 5 g of rice was weighed separately in a borosilicate crystallization capsule (diameter 6 cm), and they were inoculated with 1 mL of *B. cereus* vegetative cells at a concentration of 10^7^ CFU/g. The control samples were prepared exactly the same as the treatment samples for both spores and vegetative cells in the stationary phase, but they were directly plated without undergoing any plasma treatment.

After each plasma treatment, the samples were diluted with 10 mL of peptone water for vegetative cells and distilled water for spores. To prevent spore aggregation, vigorous shaking with glass beads was performed before taking each sample for plating. Through agitation, the *B. cereus* vegetative cells or spores that remained after treatment were extracted. Following the recovery of the solution, two sets of serial decimal dilutions (Series A and B) were prepared in duplicate. From each decimal solution, 100 μL was plated in duplicate on nutrient agar (Scharlab, Barcelona, Spain) enriched with 1g/l starch (Scharlab, Barcelona, Spain) and incubated for 18–20 h at 30 °C. After the incubation period, a manual count of *B. cereus* colonies was conducted.

### 2.6. Modeling

Experiments were performed in triplicate with two replicas per count. The experimental results were computed in Microsoft Excel by applying the Log_10_ of the survival fraction (LogS), as calculated by Equation (1).

Log(S) = N/N_0_ (1)

where N is the bacterial concentration (CFU/mL) at time t (min), and N_0_ is the initial bacterial concentration (CFU/mL) (t_0_). These data were plotted using the OriginPro software (Version 2023b. OriginLab Corporation, Northampton, MA, USA).

The obtained mean data were fitted to the Weibull survival function (2) using the GInaFiT (Version 1.8-Microsoft Office 365 copyrighted by the Katholieke Universiteit Leuven KU Leuven, Leuven-Belgium) program. This non-linear regression is a powerful technique for modeling microbial inactivation as it allows for the simultaneous obtention of a and b parameters from the survival curves.

Log_10_(N) = Log_10_(N_0_) − ((t/a)^b) (2)

where N is the microbial concentration after treatment, N_0_ is the initial microbial concentration before treatment, t is the treatment time (min), “a” is the scale parameter, and “b” is the shape of the parameter.

#### Accuracy Factor

The model was validated by calculating the accuracy factor (AF) of the experimental data with respect to predicted data by the model. This factor provides a measure of the average precision of the estimates and is given by Equation (3). The accuracy factor must always be greater than or equal to one, and the value is one if there is perfect agreement between all predictions and observed values [20].

AF = 10^(Σ|log (predicted/observed)|)/n(3)

where n is the number of observations used in the calculation.

### 2.7. Statistical Analysis

The statistical differences in the parameters obtained were determined with an analysis of variance (ANOVA) (*p*-value < 0.05), and intergroup differences were determined using Fisher’s test (LSD), which identified homogeneous subsets of means that did not differ from each other. This analysis was carried out on the mean data of parameter a for each power and matrix, using Statgraphics Centurion XIX Software (version 19.6.03 copyrighted by Statgraphics Technologies, Inc. New York, NY, USA).

## 3. Results and Discussion

**Figure 1:**
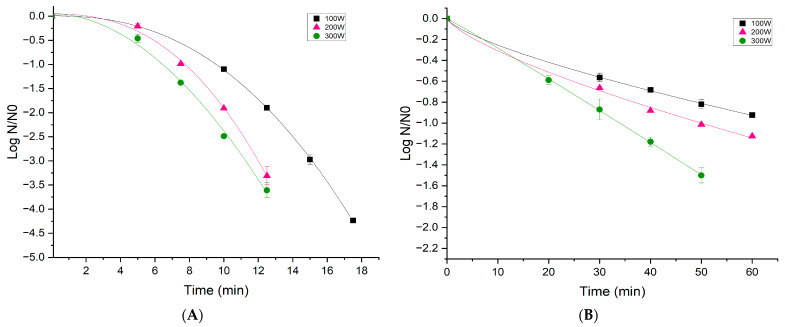
Weibull distribution function fit for *B. cereus* vegetative cells (**A**) and *B. cereus* spores (**B**) within rice grains. Inactivation as a function of power (100 W (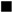), 200 W (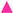), and 300 W (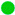)). The icons show the experimental values and the line predictions obtained by the model.

**Figure 2:**
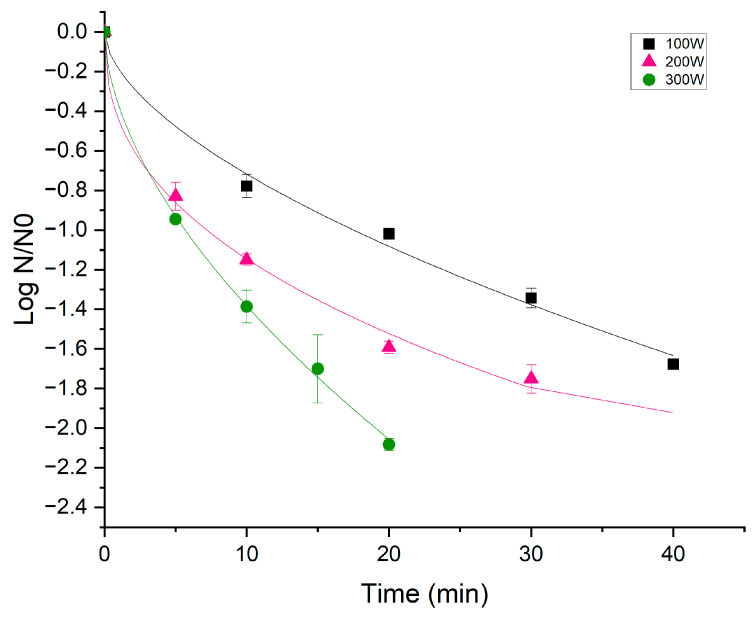
Weibull distribution function fit for *B. cereus* spores within an inert matrix (borosilicate glass slides). Inactivation as a function of power (100 W (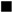), 200 W (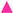), and 300 W (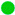)). The icons show the experimental values and the line predictions obtained by the model.

The variation in *B. cereus* response is shown in Figure 1A,B and Figure 2. A comparison was conducted among different treatment powers in various scenarios: Figure 1A shows the *B. cereus* inactivation of vegetative cells inoculated in a rice matrix; Figure 1B shows the inactivation of *B. cereus* spores inoculated in a rice matrix; and Figure 2 shows the inactivation of *B. cereus* spores spread on a borosilicate glass slide. These distinct scenarios provide insights into the varied effects of treatment powers on different microbial forms and matrices, contributing to a comprehensive understanding of the cold plasma treatment outcomes.

### 3.1. Effect of Low-Pressure Cold Plasma on B. cereus Vegetative Cells Inoculated in a Rice Matrix

Figure 1A illustrates the effect of plasma technology on *B. cereus* vegetative cells within a rice matrix after plasma treatment with different times and powers, using O_2_ as an ionization gas. As the treatment duration and intensity increased, a corresponding decrease in *B. cereus* vegetative cell counts was observed. For all power levels, the bacterial reductions achieved were close to four logarithmic cycles, reaching the detection limit of the method. Specifically, they ranged from 1.0 to 4.23 log CFU/mL with treatment times of 10 to 17.5 min at 100 W, from 0.2 to 3.3 log CFU/mL, with times of 5 to 12.5 min at 200 W, and from 0.46 to 3.61 log CFU/mL with times of 5 to 12.5 min at 300 W.

These results can be attributed to the effect of the reactive species generated by the plasma, such as reactive oxygen and nitrogen species identified as O_2_, O_3_, O_2_^−^, N, N_2_, and as a consequence of the moisture present in OH and H_2_O_2_ (spectra not shown in this document). These reactive species were formed upon the collision of charged particles with the background gas or moisture in the substrate or microorganism, leading to the disintegration of cell walls and membranes through oxidation, along with the generation of volatile components like CO, CO_2_, H_2_O, and deep-etching channels; as a consequence, direct permeabilization of the membrane cell wall caused the leakage of cellular components (potassium, nucleic acid, and proteins), the denaturation of proteins, and DNA damage. Therefore, higher plasma exposure and higher treatment potency correlated with higher decontamination [9,21,22,23,24,25].

Choi et al. [18] studied the effect of plasma on a dried black-mouth angler, observing a slightly slower decline in *B. cereus* vegetative cells when applying atmospheric DBD plasma for 30 min at 120 W, resulting in a reduction of 1.06 log cycles. Jeon et al. [26] reported a decrease of only 0.96 log CFU/g in *B. cereus* vegetative cells in red pepper powder after treatment with atmospheric DBD plasma for 20 min at 120 W. In contrast, the results found in the present study are similar to those reported by Lee et al. [27] in brown rice. They investigated the impact of atmospheric plasma DBD on *B. cereus* vegetative cells in cooked white and brown rice and found that when the treatment time was increased to 20 min at 250 W, it led to the reduction of nearly five log cycles in cooked white rice. However, smaller reductions (1.9 log cycles) were found in brown rice, showing variations in the plasma effectiveness depending on the matrix. In the same sense, Y. H. Kim et al. [17] found that treatment with atmospheric DBD plasma had different effects on *B. cereus* and *E. coli*, depending on the matrix. They found a reduction of 1 log CFU/mL in *B. cereus* after a 15 min treatment at 31 kW, while *E. coli* showed a reduction of 2 log CFU/mL when contained in a culture medium, but when *B. cereus* and *E. coli* were within red pepper powder, only a 1 log CFU/mL reduction in *E. coli* was observed, and no significant reduction in *B. cereus* was observed even with a longer treatment duration.

This variability in the results obtained by different studies could be attributed to the fact that the efficacy of plasma is significantly impacted by the nature of the matrix. Additionally, other factors, such as the microbial strain or type of microorganism and the distribution of cells within the product, play key roles. These factors can significantly influence the outcome, affecting the observed resistance or susceptibility of microorganisms to different treatments or conditions. Understanding and controlling these variables is crucial for ensuring the accuracy and reliability of microbial assays in various applications, including food safety, pharmaceuticals, and environmental monitoring.

### 3.2. Effect of Cold Plasma at Low Pressure on B. cereus Spores Inoculated in an Inert Matrix and in a Rice Matrix

As mentioned above, *B. cereus* is a bacterium with the ability to form spores, which represents the real threat of this bacterium. As it is a bacterium found in the soil, it can become the main contaminant of rice crops. *B. cereus* spores present in rice grains can resist traditional cooking methods and pose a health risk to consumers [28]. Hence, this study also aimed to examine the effect of plasma on *B. cereus* spores, both when they are spread in an inert matrix (borosilicate glass slides) and when they are inoculated in a food matrix, such as rice.

Figure 2 shows *B. cereus* spores’ inactivation in an inert matrix. It was observed that as the treatment time and power increased, the inactivation of *B. cereus* spores increased; thus, with a treatment time of 20 min and 300 W, there was a reduction in two logarithmic cycles. J. E. Kim et al. [29] found similar results since they observed that increasing the duration of the treatment produced greater inactivation of *B. cereus* spores. A significant increase in the mortality rate was observed, ranging from 1.5 ± 0.1 to 2.1 ± 0.2 log spores/cm² as the treatment time increased from 30 to 40 min. Liu et al. [19] also observed similar behavior, noting that with atmospheric Jet plasma, inactivation increased as the treatment power increased when the matrix containing the spores was inert. But, the effectiveness of their treatment was lower than the present study since they only managed to inactivate 0.99 log CFU/mL with 600 W, 2.17 log CFU/mL with 800 W, and 2.37 log CFU/mL with 1000 W when *B. cereus* spores were in Petri dishes. This could be attributed to the low-pressure conditions used in this study, where the mean free path between gas particles was extended. As a result, electron acceleration dominated in the electric field over elastic collisions with heavy particles, which would have otherwise heated the background gas. Furthermore, the use of Dielectric Barrier Discharge (DBD) equipment guaranteed more uniform treatment throughout the sample [22].

In general, the inactivation of *B. cereus* spores by plasma may be related to the effect of reactive species generated by plasma. Bacterial endospores, with their multiple layers of resistance, are difficult to remove. However, plasma power can increase electron density and the concentration of reactive species, such as atomic oxygen and hydroxyl radicals, causing damage to the cell wall, inducing electrostatic stress and morphological changes, while UV radiation contributes to cell nucleus damage, specifically causing the formation of thymine dimers in DNA [11,19,21]. While these potential effects of plasma on spores have not been fully confirmed and require further study, some of these effects have been supported by Van Bokhorst-van De Veen et al. [30]. They morphologically analyzed *B. cereus* spores after exposure to atmospheric plasma. In this case, they observed severe physical damage to the spores, which was evident in the irregular surfaces. Spores treated with plasma showed a reduction in viable counts of 0.8 log_10_ after 20 min. An interesting finding was that, when examined under a microscope, the spores transitioned from the bright to the grey phase, indicating limited water and nutrient entry. These plasma-induced grey-phase spores were unable to grow into vegetative cells in BHI media.

Figure 1B shows *B. cereus* spores’ inactivation in a rice matrix. In this case, with a treatment time of 60 min and a power of 100 W, a reduction of almost one logarithmic cycle was achieved. Similarly, at 60 min with 200 W of power, a reduction of 1.1 logarithmic cycles was attained, and with a power of 300 W for 50 min, there was a reduction of 1.4 logarithmic cycles. The results show that the inactivation reached in a food matrix is lower than the one obtained in the inert surface. This could be for diverse reasons. The rice grain has a porous surface, and this porosity may have increased as a consequence of the plasma’s effect on the rice grain’s surface. It is well documented that plasma treatment has the capability to modify surfaces [24,31]. Consequently, the microorganism may have become trapped in these pores, which could be another reason why plasma is less effective on rice grains compared to inert surfaces. Beyrer et al. [32] used *B. coagulans* spores to investigate how spore loading on flat glass carriers or when mixed in the aqueous phase with a non-soluble powder affects the efficiency of inactivation by a DBD CAP. The inactivation effect for spores directly exposed to DBD CAP on flat glass carriers (reference value) was clearly reduced by native rice starch granules (the non-porous powder model system) and shells of diatoms (the highly porous dust model system). The granules are associated with significant protective effects, probably due to the shading of UV light and structural modification of starch and zein powder. An alternative explanation is based on the erosion effect on particles, in general, and spores, more specifically, which are less exposed to UV effects than those on non-inert surfaces. In conclusion, pores in shells of diatoms can protect microorganisms a magnitude better than starch particles with smoother surfaces, which are less protective for spores against cold plasma and inert surfaces. The matrix effect on the spore’s inactivation was also noted by Liu et al. [19]. When the spores were inoculated on dry pepper grains, only a reduction of 1.37 log CFU/g was obtained using a power of 800 W for 20 min. The matrix effect can be associated with rice medium; cells can elongate and form non-spherical microcolonies that resemble structured spaghetti strands, as observed in the study by Warda et al. [33]. These structures pose challenges for removal from adhered surfaces due to their viscous nature. Furthermore, the abundant nutrients and other organic components of food contribute to the formation of these *B. cereus* spore aggregates. As a result, when exposed to plasma treatment, the outer layer of the spore becomes more resistant to the treatment.

Therefore, the results of this study agree with the findings of other researchers, suggesting that the matrix effect plays a relevant role in the response of *B. cereus* spores to plasma treatment. It can be said that, as in other food processes, the food matrix, considering the pH, aw, composition, etc., plays an important role in the inactivation of microorganisms or bacterial spores due to its complex structure and the formation of aggregates. This poses additional challenges for effective spore removal, especially because rice is a highly porous granulated matrix that could have two effects; on the one hand, it can shield ultraviolet light, and on the other, it can protect bacterial spores in its pores, so the net effect of the cold plasma is less compared to on the inert surface where the spores are arranged in a monolayer. Although more research is required to fully understand these effects of plasma, preliminary studies have shown significant physical damage to the spores, suggesting a reduction in their viability and ability to develop into vegetative cells. These findings underscore the importance of considering the matrix effect when designing plasma treatment strategies to ensure effective decontamination in various food matrices.

### 3.3. Low-Pressure Cold Plasma Power Effect on the Inactivation of B. cereus at a Specific Time

To further investigate the effect of plasma as a function of treatment intensity (treatment power), a specific treatment time was established to evaluate the inactivation of *B. cereus* spores dispersed on an inert matrix (borosilicate glass slides) and inoculated in a rice matrix as well as the vegetative cells of *B. cereus* inoculated in a rice matrix. The chosen treatment times were 30 min for the spores and 12.5 min for the vegetative cells. These durations were selected based on an analysis of variance (ANOVA) conducted with the experimental data obtained, which indicated that these treatment durations were able to reveal the impact of power on the inhibition of *B. cereus.*

Treating vegetative cells of *B. cereus* in a rice matrix for 12.5 min resulted in a reduction of 1.9 to 3.31 UFC/mL when increasing the power from 100 W to 200 W. However, no further significant logarithmic reduction (*p* > 0.05) was observed when increasing the power from 200 W to 300 W (3.31 to 3.61 UFC/mL, respectively). On the other hand, as far as spores in a rice matrix are concerned, it is necessary to increase the treatment power up to 300 W to obtain significant differences in spore inactivation (0.87 CFU/mL), while in an inert matrix (borosilicate glass slides), there is a significant logarithmic reduction increase with power (1.34, 1.75, and 2.59 CFU/mL for 100 W, 200 W, and 300 W, respectively). This effect on bacterial spores could be attributed to the fact that the increase in treatment power leads to a higher density of reactive species. This high-power density can also contribute to the breakage of disulfide bonds in the protein coat of spore cells [12,34]. These chemical and structural changes may make the spores more susceptible to the attack of reactive species or excited molecules [19,21].

Based on the results obtained in this research, it can be observed that the use of low-pressure cold plasma has a significant effect on the reduction in both vegetative cells and the spores of *B. cereus*. It was observed that the power intensity and the type of matrix are critical variables that affect the effectiveness of the treatment more than time. These observations suggest that higher power densities may improve treatment efficacy by promoting chemical and structural changes in spores, making them more susceptible to reactive species. This highlights the importance of optimizing the energy settings to achieve effective microbial inhibition in different matrices. Despite less effective spore inactivation compared to vegetative cells when present in a rice matrix, it suggests the possibility of using this technology as a non-thermal disinfection method before rice processing. Reducing the initial *B. cereus* spore load in the dry rice grain could help make subsequent rice cooking procedures more efficient. For example, if combined with other technologies such as microwaves [29], as explored in previous studies, or with natural antimicrobials, as investigated in other works [6,35], it is possible to obtain ready-to-eat food products that meet safety standards.

### 3.4. Modeling the Impact of Low-Pressure Cold Plasma on B. cereus

In this study, the Weibull model was employed to characterize inactivation parameters using experimental data from both vegetative cells and the spores of *B. cereus* treated with low-pressure cold plasma sterilization. While initially proposed by Mafart et al. [36] and Peleg & Cole [37] for thermal sterilization, this model was also successfully applied by Valdez-Narváez et al. [38] in previous research that investigated thermal treatment combined with natural antimicrobials for *B. cereus* inactivation, and it has demonstrated good performance when microbial inactivation is modeled by non-thermal treatments, such as high hydrostatic pressures and pulsed electric fields. Therefore, it is interesting to determine if this model is also a good candidate for representing inactivation with other non-thermal technologies, such as cold plasma.

For a specific condition, the average experimental inactivation data were fitted to the Weibull model (Equation (2)) to obtain the scale parameter “a” and shape parameter “b”. The “a” parameter serves as an indicator of treatment resistance, representing *B. cereus’* survival capability under various treatment conditions. On the other hand, the “b” parameter reflects the curve’s shape, indicating the relationship between treatment resistance and time. A “b” value > 1 suggests a concave (upward) curve, a value equal to 1 indicates a linear curve, and a value < 1 represents a convex (downward) curve.

The results (Table 1) showed that the Weibull model fitted the experimental data favorably, providing insights into the effect of cold plasma in different study scenarios. The survival curves exhibited different shapes, as depicted in Figure 1A,B. When dealing with *B. cereus* vegetative cells in a rice matrix (Figure 1A), the curves displayed a shoulder region (gentle initiation). The minimum time required to cause damage to bacterial cells is reflected by the shoulder region, and it can be observed that the mortality rate is time-dependent with “b” values greater than one. On the other hand, *B. cereus* spores’ inactivation, as shown in Figure 1B and Figure 2, was characterized by inactivation curves with convex shapes (“b” values < 1), suggesting the existence of a residual spore fraction resistant to plasma treatment.

After statistical analysis for all the samples and combinations studied, as plasma power increased, a significant decrease (*p*-value < 0.05) in the parameter “a” was confirmed, meaning that *B. cereus* resistance decreases as treatment intensity increases. However, there are significant differences between “a” values for vegetative cells and spores, with the latter being more resistant to treatment, as previously mentioned by Liu et al. [19] and Bourke et al. [21]. At the same time, significant differences were also found between “a” values for spores depending on the matrix, denoting spores’ higher resistance in rice than on the inert surface.

As indicated before, the food matrix acts as an additional protective barrier for spores. Comparing the “a” value from Table 1, it is evident that it significant decreases when spores were contained on an inert surface. For instance, at 100 W power, the “a” value was 17.72 for spores on slides and 68.17 for spores on rice. At 200 W, the “a” value for spores on slides was 7.57, and for spores on rice, it was 49.69. When treated with 300 W, the “a” value was 5.90 for spores on slides and 34.05 for spores on rice. This significant difference in “a” value and the shape of the curve detailed above confirms what was previously observed with the inactivation data in that the matrix has a substantial impact on the treatment; when the spores are contained in the food matrix, they adhere to rice grains, making it more challenging for plasma to penetrate the matrices and have a reduced effect on the spores.

Regarding methods for fitting cold plasma effects, several authors have found that the Weibull model effectively represents the impact of plasma on bacterial inactivation. Qian et al. [39] studied the effect of plasma on Listeria monocytogenes and Salmonella enteritidis with three kinetic models with which they found that the Weibull model had a good fit, demonstrating its suitability for describing the inactivation kinetics of these pathogens under plasma treatment conditions. Kim et al. [29] also used the Weibull model to study the effect of plasma on B. cereus, obtaining values of “a” at 20.66 and “b” at 0.41 after 40 min of treatment at 400 W, while Hertwig et al. [40] also used a Weibull model to study the effect of plasma, obtaining values of “a” at 2.35 and “b” at 1.10 for B. atrofaeus and for B. subtilis values “a” at 7.40 and “b” at 4.27. These results differ from those in this study, which could be attributed to variations in the matrix or treatment equipment.

Finally, the model was validated by calculating the accuracy factor (AF) using Equation (3). For B. cereus vegetative cells within a rice matrix, the AF value was 1.033, indicating a prediction error rate of 3.3%. For B. cereus spores within the same matrix, the AF value was 1.021, corresponding to a 2.1% prediction error rate. For B. cereus spores on a borosilicate slide, the AF value was 1.009, reflecting a prediction error rate of 0.9%. This model effectively predicts the resistance of B. cereus vegetative cells and spores on both rice and borosilicate glass slides after exposure to various plasma treatments.

These data provide a comprehensive understanding of the effects of cold plasma application at different stages of the B. cereus life cycle and within diverse matrices. Modeling the kinetics of microbial inactivation is crucial to predict how the microbial population changes with time and treatment conditions, allowing scale-up at an industrial level to optimize disinfection processes, validate food preservation methods, design control strategies of contamination, and comply with food safety standards.

## 4. Conclusions

This study demonstrates that using a low-pressure cold plasma system with O_2_ as the ionization gas can reduce both the vegetative cells and spores of *B. cereus* in both a rice matrix and an inert surface. However, despite achieving less spore inactivation compared to vegetative cells when contained in a rice matrix, this could be promising for the use of this technology as a non-thermal disinfection technique prior to processing/cooking low-water activity matrices such as rice. Plasma treatment has shown its ability to reduce the initial *B. cereus* spore’s load, so the knowledge gained in this study highlights the potential industrial applications of cold plasma technology in food processing. By integrating cold plasma treatments as a pre-processing step, food manufacturers can improve the microbial safety of products, extending their shelf life and ensuring consumer health and safety. This non-thermal disinfection method offers a viable alternative; however, seeing the impact that the matrix has on the treatment, further research is necessary to optimize and industrially scale this technology in various food matrices.

## Figures and Tables

**Table 1:** Weibull survival function parameters obtained by fitting experimental mean data.

	100 W				200 W				300 W			
	*a*	*b*	R^2^_adj_	MSE	*a*	*b*	R^2^_adj_	MSE	*a*	*b*	R^2^_adj_	MSE
RV	9.54 ± 0.26 *^, a^	2.39 ± 0.10	0.9997	0.0008	7.59 ± 0.10 *^, b^	2.43 ± 0.13	0.9951	0.0091	6.28 ± 0.20 *^, c^	1.91 ± 0.15	0.9914	0.0188
RS	68.17 ± 0.04 *^, A, a^	0.71 ± 0.07	0.9994	0.0001	49.69 ± 0.04 *^, A, b^	0.73 ± 0.03	0.9950	0.0010	34.05 ± 1.50 *^, A, c^	1.04 ± 0.04	0.9995	0.0002
BS	17.72 ± 0.10 ^B, a^	0.60 ± 0.02	0.9870	0.0053	7.57 ± 0.07 ^B, b^	0.41 ± 0.02	0.9917	0.0040	5.90 ± 0.90 ^B, c^	0.58 ± 0.02	0.9978	0.0014

RV: Rice with vegetative cells. RS: Rice with spores. BS: Borosilicate glass slides with spores. The asterisk (*) denotes statistically significant differences between the vegetative cells and spores of *B. cereus* within a rice matrix, while capital letters (A and B) denote statistically significant differences between *B. cereus* spores within distinct matrices. Lowercase letters (a, b, and c) denote statistically significant differences between the various study groups for each power level. ANOVA (*p* < 0.05).

## Data Availability

The original contributions presented in the study are included in the article, further inquiries can be directed to the corresponding author.
